# Mechanisms of Soil Microbial Community Adaptation in Cold-Region Wetlands Under Retrogressive Succession

**DOI:** 10.3390/life15050817

**Published:** 2025-05-20

**Authors:** Junnan Ding, Shaopeng Yu

**Affiliations:** Heilongjiang Province Key Laboratory of Cold Region Wetland Ecology and Environment Research, Harbin University, Harbin 150086, China; wetlands1972@126.com

**Keywords:** retrogressive succession, wetland degradation, soil microorganisms, community structure, high-throughput sequencing, soil physicochemical properties

## Abstract

Retrogressive succession alters soil conditions and microbial community dynamics in cold-region wetlands, yet its ecological implications remain understudied. This study explored the structure and function of soil microbial communities across three successional stages: swamp (SP), swamped meadow (SM), and meadow (MW). High-throughput 16S rRNA gene sequencing identified 2852 operational taxonomic units (OTUs), with 1682 shared among all stages (58.85%). Alpha diversity indices, including Shannon, Chao, ACE, and Sobs, were significantly higher in MW, with the Shannon index increasing by approximately 32% compared to SP, indicating enhanced richness and evenness. In contrast, Simpson and Coverage indices were highest in SP. Proteobacteria, Actinobacteriota, and Acidobacteriota were dominant phyla, showing distinct distributions across stages. Beta diversity analysis (PCoA and NMDS) revealed clear separation of microbial communities. Soil organic carbon (SOC), pH, soil water content (SWC), cation exchange capacity (CEC), and bulk density (BD) significantly influenced microbial composition and distribution. Functional prediction using FAPROTAX and BugBase indicated a shift from anaerobic metabolism, nitrogen fixation, and cellulolysis in the SP to aerobic chemoheterotrophy and stress tolerance in MW. These results demonstrate that microbial communities adapt to changing soil environments during retrogressive succession, highlighting their role in ecosystem function and resilience in cold-region wetlands.

## 1. Introduction

Global climate change and intensified human activities have imposed severe degradation pressures on wetland ecosystems [1]. Wetlands, covering approximately 6% of the Earth’s land surface, are unevenly distributed across different continents, with the largest concentrations found in North America, Northern Europe, and Asia, particularly in Canada, Russia, and China [2]. These ecosystems play an indispensable role in regional water resource regulation, biodiversity conservation, and serving as ecological safety barriers, and their functionality is increasingly drawing widespread attention [3]. Existing studies have primarily focused on wetland degradation and retrogressive succession in high-altitude or plateau regions, emphasizing aspects such as carbon and nitrogen dynamics, wetland classification, and degradation mechanisms [4]. However, systematic investigations on the changes in soil microbial community structure and function during retrogressive succession in low-altitude cold-region wetlands, particularly in northeastern China, remain scarce, thereby limiting a comprehensive understanding of the overall degradation process and the underlying mechanisms of ecosystem functional loss [5,6].

In cold-region wetland ecosystems, soil microorganisms are key regulators of essential ecological processes such as soil organic carbon (SOC) decomposition, nutrient transformation, and energy flow [7]. Recent studies employing high-throughput sequencing techniques have revealed significant changes in microbial community structure and function during wetland degradation [8]. It has been shown that the transition from pristine marshes to swamp meadows and further to meadows is accompanied by marked changes in soil physicochemical properties, which in turn significantly affect microbial diversity and community composition, ultimately influencing overall ecosystem function [9]. Moreover, mechanistic studies indicate that the internal processes underlying microbial responses to environmental changes during retrogressive succession involve several interrelated factors [10]. As wetland degradation progresses, declines in SOC, fluctuations in soil moisture, and reductions in key nutrients such as nitrogen, phosphorus, and potassium emerge as critical environmental drivers [11]. Concurrently, variations in soil cation exchange capacity (CEC) affect the retention and availability of these nutrients, thereby directly influencing microbial growth and metabolism [12]. For example, decreased nutrient availability may intensify competition among microbial populations, favoring species that possess efficient nutrient uptake mechanisms and can thrive under low-nutrient conditions, while the reduction in SOC limits the microbial capacity to utilize organic matter, consequently impacting the dynamics of organic matter decomposition and nutrient cycling [13,14]. Additionally, changes in soil moisture not only directly influence the microbial habitat but also alter soil redox conditions, affecting the balance between aerobic and anaerobic microbial processes [15]. The interplay between fluctuations in soil pH and CEC further modulates nutrient solubility and availability, thereby exerting comprehensive regulatory effects on microbial metabolic activity and community composition [16]. Collectively, these physicochemical indicators and microbial community dynamics interact in complex and close ways that determine changes in ecosystem function during wetland degradation [17]. However, systematic studies that integrate microbial community profiling with detailed analyses of SOC, soil moisture, key nutrients (nitrogen, phosphorus, potassium), and CEC in low-altitude cold-region wetlands remain scarce [18]. Such integrated research is of great importance for comprehensively understanding the mechanisms underlying microbial responses to environmental changes and the inherent processes leading to ecosystem functional decline during wetland degradation [19].

The Heilongjiang Raohe National Nature Reserve, located in northeastern China within a cold-temperate transitional zone, represents an important region for studying degradation in low-altitude cold-region wetlands. The reserve is characterized by abundant wetland resources, predominantly marshes and swamp meadows, with distinct spatial and temporal gradients in wetland landscapes that make it an ideal study area for retrogressive succession. The Raohe region not only provides a stable habitat for regional flora and fauna but also plays a critical role in maintaining hydrological balance and ensuring ecological safety. Currently, research on the microbial community structure and function in the context of the transition from marshes to swamp meadows and then to meadows within this region remains fragmented, highlighting the urgent need for systematic field surveys and experimental analyses to elucidate the intrinsic patterns of microbial responses to environmental changes during degradation [20].

To explore microbial community dynamics along a degradation trajectory in cold-region wetlands, we employed a space-for-time substitution (SFT) approach, a widely recognized method in ecological research that infers temporal processes from spatial gradients when long-term data are unavailable or impractical to obtain [21]. This technique is particularly suitable for succession studies, provided that the selected sites share similar climatic and edaphic conditions and reflect a unidirectional change in ecosystem state [22]. Three representative stages of retrogressive succession were selected in this study, namely swamp (SP), swamped meadow (SM), and meadow (MW), all located within the Raohe National Nature Reserve. Site selection was based on consistent hydrological regimes, geomorphological continuity, and vegetation zoning patterns, with minimal anthropogenic disturbance. Soil physicochemical properties were measured, and high-throughput sequencing was used to analyze the structure and function of soil microbial communities. The specific research objectives are as follows: (1) to analyze the spatiotemporal variation of soil microbial community structure and functional characteristics during wetland retrogressive succession and to examine the driving effects of soil physicochemical parameters on microbial community shifts; (2) to elucidate the response mechanisms of microbial communities in maintaining ecosystem function during wetland degradation, thereby providing empirical data for a systematic understanding of degradation mechanisms in low-altitude cold-region wetlands. Achieving these objectives is expected to offer a scientific basis for wetland degradation monitoring, ecological restoration, and regional ecological management, and to provide new theoretical support for the sustainable utilization and conservation of global wetland ecosystems.

## 2. Materials and Methods

### 2.1. Site Description

**Figure 1:**
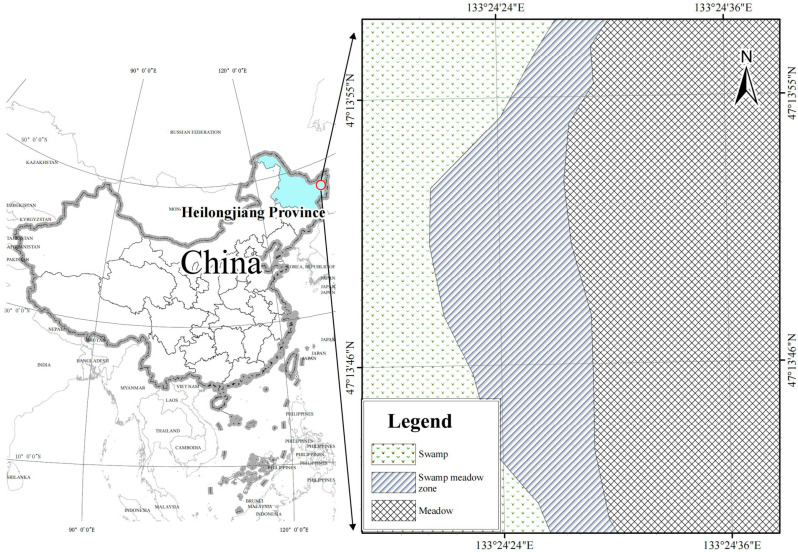
Map showing different vegetation locations of the study. Abbreviations: SP, swamp; SM, swamped meadow; MW, meadow.

Situated in the Raohe National Nature Reserve in Heilongjiang Province, northeastern China (48°45′–49°00′ N, 133°30′–133°50′ E), the study area is a well-preserved low-altitude cold-region wetland ecosystem exhibiting a distinct environmental gradient from marshes to swamp meadows and finally to meadows (Figure 1). The elevation across the sampling sites ranges from approximately 63.5 to 68.4 m above sea level. The region experiences a cold temperate continental monsoon climate, with an average annual temperature of around 1 °C, a frost-free period of about 90 days, and annual precipitation of roughly 600 mm, predominantly during summer months.

Dominant soil types include hydric soils such as peat and gley soils, which are essential for nutrient cycling and carbon sequestration. Hydrological conditions vary substantially along the successional gradient: the swamp (SP) zone remains permanently saturated with groundwater close to or slightly above the soil surface; the swamped meadow (SM) zone experiences seasonal saturation, with the water table typically 0–10 cm below the surface; the meadow (MW) zone is well-drained with water tables more than 20 cm below the surface, supporting more aerobic soil conditions. Detailed geographic and environmental metadata for the three successional stages include sampling coordinates, elevation, water table depth, and hydrological conditions.

In the marsh zone, hydrophytic vegetation adapted to prolonged waterlogging prevails, with typical species including *Phragmites australis*, *Typha angustifolia*, and *Carex appendiculata*. The swamp meadow zone represents a transitional stage, characterized by a mixed community with species such as *Calamagrostis angustifolia*, *Carex dahurica*, and *Sparganium stoloniferum*, where fluctuating water levels influence both nutrient availability and microbial activity. In the meadow zone, reduced hydric conditions favor the dominance of terrestrial grassland species such as *Potentilla anserina*, *Festuca arundinacea*, and *Deschampsia cespitosa*, with drier, more aerated soils, leading to distinct shifts in microbial community structure and soil function [23,24].

### 2.2. Sample Collection

In September 2024, soil samples were collected from three designated sampling sites within the study area. At each site, 12 soil samples were obtained from the 0–20 cm topsoil layer using an S-shaped pattern to capture spatial heterogeneity, resulting in a total of 36 soil samples. Plant roots, litter, stones, and other impurities were carefully removed during collection. All 36 samples were treated and analyzed individually to retain site-level resolution. After field collection, samples were bagged, labeled, and stored in an icebox at approximately 4 °C before being transported to the laboratory.

In the laboratory, approximately 10 g of fresh soil from each individual sample was transferred into a sterile 50 mL Falcon tube and stored at −80 °C for microbial analysis. The remaining portion of each sample was air-dried, sieved through a 2 mm mesh, and homogenized separately for physicochemical analyses. All soil physicochemical parameters including pH, SOC, TN, TP, TK, SWC, CEC, and BD were measured in triplicate (*n* = 3 per sample) to ensure analytical accuracy and reproducibility, while preserving the integrity of individual sample resolution.

### 2.3. Analysis of Soil Physicochemical Properties

Soil physicochemical properties were analyzed following standard laboratory procedures. Soil pH was measured using a pH meter (SevenCompact S220, Mettler Toledo, Greifensee, Switzerland), calibrated with standard buffer solutions (pH 4.0, 7.0, and 10.0). Soil organic carbon (SOC) content was determined using the Walkley–Black titration method with potassium dichromate and sulfuric acid reagents (Sinopharm Chemical Reagent Co., Ltd., Shanghai, China) [25]. Soil moisture content was assessed via the gravimetric method by drying a known mass of soil at 105 °C in an oven (DHG-9075A, Shanghai Yiheng Scientific Instrument Co., Ltd., Shanghai, China) until a constant weight was achieved. Total nitrogen (TN) was quantified using the Kjeldahl method after digestion with concentrated H_2_SO_4_ (Aladdin Biochemical Technology Co., Ltd., Shanghai, China) [26]. Total phosphorus (TP) was determined through HClO_4_–H_2_SO_4_ digestion followed by molybdenum–antimony colorimetry using a UV-Vis spectrophotometer (UV-2600, Shimadzu Corporation, Kyoto, Japan) [27]. Total potassium (TK) was measured after digestion with a mixture of nitric acid (HNO_3_), perchloric acid (HClO_4_), and hydrofluoric acid (all from Sinopharm Chemical Reagent Co., Ltd., Shanghai, China), followed by analysis with a flame photometer (FP640, Shanghai Precision Scientific Instrument Co., Ltd., Shanghai, China) [28]. Soil bulk density (BD) was determined using the core method; undisturbed soil samples were collected using a stainless-steel core sampler (Eijkelkamp Soil & Water, Giesbeek, Netherlands), oven-dried at 105 °C, and BD was calculated by dividing the dry mass by the volume of the core [29]. The cation exchange capacity (CEC) was measured using the ammonium acetate extraction method at pH 7.0, according to standard procedures, and quantified using an atomic absorption spectrophotometer (AA-7000, Shimadzu Corporation, Kyoto, Japan) [30].

### 2.4. DNA Extraction and High-Throughput 16S rRNA Gene Paired-End Sequencing

Total genomic DNA was isolated from 0.5 g of each soil sample using the E.Z.N.A.^®^ Soil DNA Kit (Omega Bio-Tek, Norcross, GA, USA) according to the manufacturer’s instructions. The concentration and purity of extracted DNA were evaluated using a NanoDrop 2000 spectrophotometer (Thermo Fisher Scientific, Waltham, MA, USA) based on A260/A280 absorbance ratios, and DNA integrity was checked by 1% agarose gel electrophoresis. Bacterial 16S rRNA genes were amplified in a two-step PCR workflow using the GeneAmp PCR System 9700 (Applied Biosystems, Carlsbad, CA, USA). The V3–V4 hypervariable regions were targeted using the universal primer pair 515F (5′-GTGCCAGCMGCCGCGGTAA-3′) and 907R (5′-CCGTCAATTCMTTTRAGTTT-3′). The first PCR amplified the target region, followed by a second PCR to incorporate sample-specific barcodes. Each 25 μL PCR reaction included the following: 1× buffer, 1.5 mM MgCl_2_, 0.2 mM dNTPs, 0.5 μM primers, 1.25 U Taq DNA polymerase (Takara, Dalian, China), and 1 μL DNA template. Thermocycling conditions were as follows: initial denaturation at 95 °C for 3 min; 25 cycles of 95 °C for 30 s, 55 °C for 30 s, and 72 °C for 45 s; and a final extension at 72 °C for 10 min. To evaluate PCR inhibition, a subset of DNA samples was serially diluted and re-amplified to ensure consistent amplification efficiency. Amplified products were purified using a DNA Clean and Concentrator Kit (Omega Bio-Tek) and quantified with a QuantiFluor^®^-ST fluorometer (Promega, Madison, WI, USA). Purified amplicons were normalized and pooled in equimolar concentrations for downstream sequencing. The samples were then sent to Shanghai Meiji Biotechnology Co., Ltd. (Shanghai, China) for sequencing on the Illumina HiSeq 2500 PE250 platform (San Diego, CA, USA) for high-throughput sequencing [31].

### 2.5. Sequencing Data Processing and Analysis

Raw sequencing reads were subjected to quality control using the QIIME pipeline (Caporaso Lab, Northern Arizona University, Flagstaff, AZ, USA) integrated with DADA2 (Benjamin Callahan, North Carolina State University, Raleigh, NC, USA). Low-quality sequences (Phred score < Q20) and reads shorter than 200 base pairs were excluded to ensure high-fidelity data. Chimera detection and removal were carried out using the UCHIME algorithm to eliminate artificial sequences. Adapter and primer residues were trimmed using Cutadapt (version 3.4), and taxonomically unclassified or non-bacterial reads were filtered by alignment against the SILVA reference database (version 138). To reduce potential bias from sequencing noise, singleton operational taxonomic units (OTUs)—those detected only once across the dataset—were removed prior to downstream analysis.

### 2.6. Statistical Analysis

Community diversity parameters (Shannon, Simpson, Coverage, Sobs, Ace, and Chao1 indices) were used to conduct alpha diversity analyses using the mothur software (version 1.44.3) [32]. Beta diversity analysis was performed using R software (version 4.2.2; R Core Team, Vienna, Austria), and differences in microbial communities were assessed using one-way analysis of variance (ANOVA) and the least significant difference test. Data were statistically analyzed using Microsoft Excel 2007 (Redmond, WA, USA) and SPSS 22.0 (IBM, Inc., Armonk, NY, USA). To evaluate the significance of beta diversity differences across successional stages, permutational multivariate analysis of variance (PERMANOVA) was performed based on Bray–Curtis distances using the “adonis” function in the vegan package (version 2.6-4) in R software (version 4.2.2). The analysis used 999 permutations. The resulting effect size (R^2^) and *p*-values were reported to assess the strength and significance of group separation. Bonferroni correction was applied to adjust for multiple pairwise comparisons where necessary. Microbial functions of the soil bacteria were predicted by FAPROTAX and FUN Guide [33]. BugBase (Bengaluru, India) was used to annotate the functions of bacteria, and the OTU table clustered by 97% sequence similarity was used as the input file. The output table was standardized by the predicted number of 16S copies, and the microbial phenotype was then predicted using the preprocessed database. The threshold was automatically selected by the BugBase tools, categorizing bacteria into groups, such as aerobic, anaerobic, stress-tolerant, Gram-negative, Gram-positive, and potentially pathogenic [34,35].

## 3. Results

### 3.1. Soil Physicochemical Properties

There were significant differences in soil physicochemical properties across different succession stages (Table 1). Soil pH ranged from 6.38 to 6.57, indicating a generally weakly acidic condition; meanwhile, there were no statistically significant differences in pH values among the groups. The contents of SOC, BD, TN, TP, and TK of the MW were significantly higher than those of the SP and SM (*p* < 0.05), and the contents of SOC of the MW were 14.00 and 19.91% higher than the SP, respectively. The contents of SWC of the SP were significantly higher than those of the SM and MW (*p* < 0.05). The contents of TN of the SP and MW were 22.74 and 28.57% higher than the SM (*p* < 0.05), respectively. The soil CEC content in the SM was significantly higher than that in both the SP and MW, with increases of 34.94 and 23.88%, respectively.

### 3.2. Microbial Alpha Diversity

**Figure 2:**
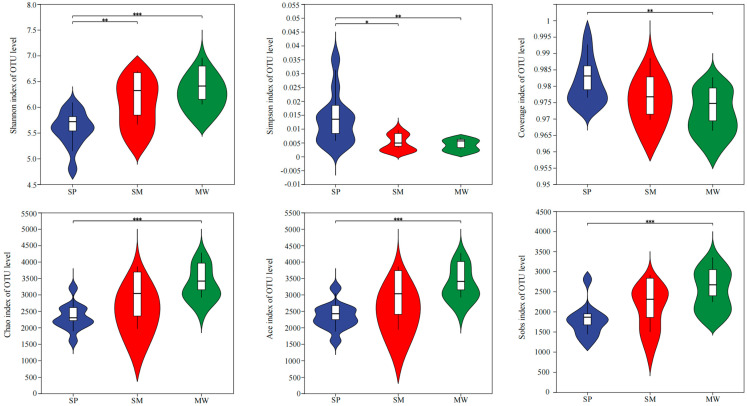
Diversity index of soil bacterial communities at the OTU level. Abbreviations: SP, swamp; SM, swamped meadow; MW, meadow. * *p* < 0.05, ** *p* < 0.01, *** *p* < 0.001.

At the OTU level, the alpha diversity index quantifies both species richness and relative abundance within a community. An analysis of bacterial and microbial diversity based on OTU-level data reveals variations in the alpha diversity index, as demonstrated by the statistical *t* tests shown in Figure 2. In MW, the Shannon, Chao, Ace, and Sobs diversity indices were significantly higher than those in the SP (*p* < 0.001), whereas the Simpson and Coverage indices were significantly higher in the SP compared to the MW (*p* < 0.01). In the SM, the Shannon diversity index was also significantly higher than in the SP (*p* < 0.01), whereas the Simpson diversity index was significantly higher in the SP compared to the SM (*p* < 0.05).

### 3.3. Species Venn Diagram and Unique OTU Analysis

**Figure 3:**
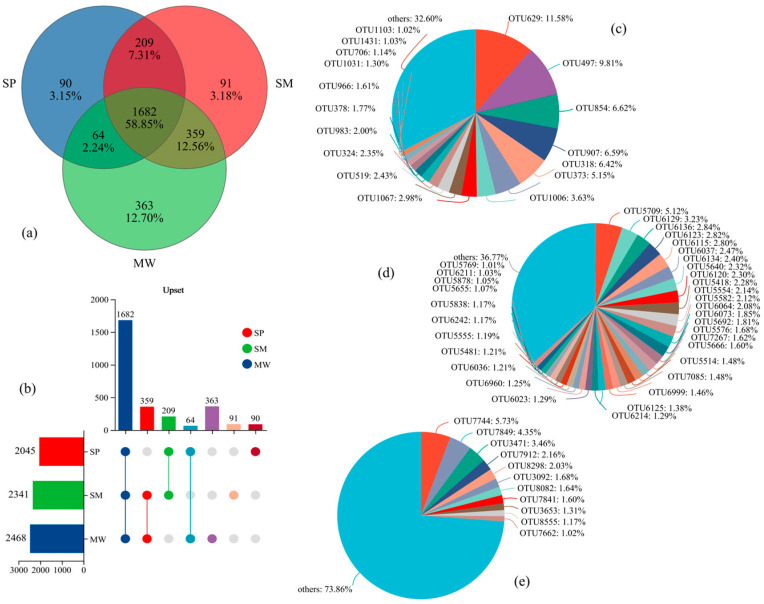
Relative abundance of soil bacterial communities in different succession stages of wetland at the level of OTU. (**a**) Venn diagram of OTU-based microbial community. (**b**) Upset diagram showing shared and unique OTUs across successional stages. (**c**) OTU-level microbial community composition in swamp (SP) soils. (**d**) OTU-level microbial community composition in swamped meadow (SM) soils. (**e**) OTU-level microbial community composition in meadow (MW) soils. Note: upset plot interpretation: the horizontal bar chart on the left indicates the total number of OTUs within each successional stage. The matrix in the middle shows the presence (dots) and intersections (connected dots) of OTUs across different stages. The vertical bars on top represent the number of OTUs that are unique to or shared among the corresponding stage combinations.

The intersection of OTU sequences exhibiting over 97% similarity with the soil bacterial communities associated with each treatment is depicted as a Venn diagram (Figure 3). A total of 2852 operational taxonomic units (OTUs) classified into 42 bacterial phyla were identified in the analyzed soil. Among the different succession stages of the wetland, 1682 OTUs were shared among all stages, accounting for 58.85% of the total OTUs. In contrast, 90 OTUs (3.15%) were unique to the SP stage, 91 OTUs (3.18%) were unique to the SM stage, and 363 OTUs (12.70%) were unique to the MW stage (Figure 3a). The abundance of bacterial OTUs was ranked as follows: MW (2468) > SM (2341) > SP (2045), (Figure 3b). In the SP stage, the OTU distribution revealed distinct dominant taxa, with OTU629 and OTU497 accounting for 11.58% and 9.81%, respectively, while the “others” category comprised a total of 32.60% (Figure 3c). In contrast, the primary OTUs in the SM stage exhibited relatively lower relative abundances; the most abundant OTU, OTU5709, accounted for only 5.12%, with the remaining key OTUs ranging from 1.01% to 3.23%, and the “others” category comprised 36.77% (Figure 3d). In the MW stage, the most abundant OTU was OTU7744 (5.73%), followed by OTU7849 (4.35%) and OTU3471 (3.46%); however, the “others” category was notably higher at 73.86% (Figure 3e).

### 3.4. Soil Bacterial Structure Analysis

**Figure 4:**
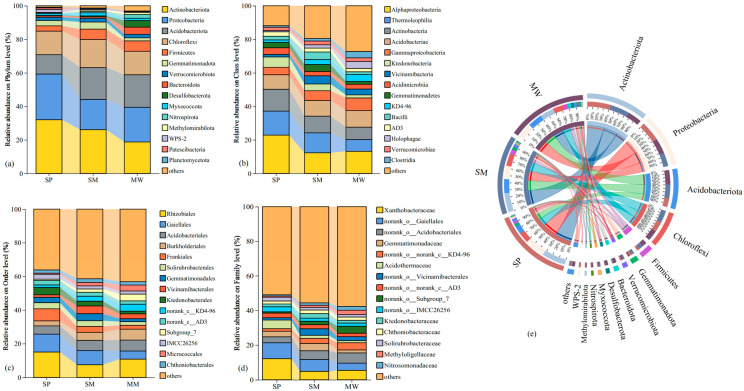
The relative abundance of phylum (**a**), class (**b**), order (**c**), family (**d**), and Circos analysis (**e**) in bacterial communities. Abbreviations, SP: swamp; SM: swamped meadow; MW: meadow.

Figure 4a shows that the phyla with an average relative abundance greater than 1% include Actinobacteria, Proteobacteria, Acidobacteria, Chloroflexi, and Firmicutes. Among these, Actinobacteria dominates the SP and SM stages, accounting for 32.01% and 26.09%, respectively, whereas Proteobacteria predominates in the MW stage with a relative abundance of 20.66%. At the class level (Figure 4b), the dominant classes are *Alphaproteobacteria*, *Thermoleophilia*, and *Actinobacteria*. Compared with the SP stage, the relative abundance of *Alphaproteobacteria* decreases by 43.83% in the SM and 42.33% in the MW, while *Thermoleophilia* decreases by 16.88% and 51.08%, respectively. At the order level (Figure 4c), *Rhizobiales*, *Gaiellales*, and *Acidobacteriales* are predominant; notably, the abundance of *Rhizobiales* decreases by 50.36% in the SM and 28.18% in the MW relative to the SP. At the family level (Figure 4d), the dominant groups are *Xanthobacteraceae*, *norank_o__Gaiellales*, and *norank_o__Acidobacteriales*. Finally, as succession progresses (Figure 4e), the relative abundance of *Actinobacteria* in the MW stage decreases by 41.42% and 28.13% compared to the SP and SM stages, respectively. Furthermore, the Proteobacteria in the SM stage decrease by 33.73% and 12.63% relative to the SP and MW stages, while the Actinobacteria in the SP stage decrease by 39.09% and 40.51% when compared to the SM and MW stages.

### 3.5. Microbial Beta Diversity

**Figure 5:**
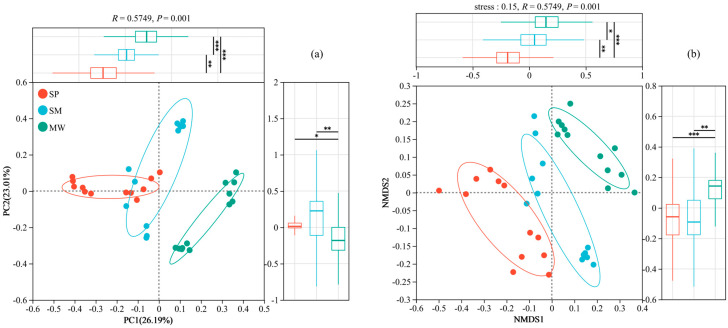
The PCoA (**a**) and NMDS (**b**) analysis of soil bacteria at the OTU level. Abbreviations: SP, swamp; SM, swamped meadow; MW, meadow. * *p* < 0.05, ** *p* < 0.01, *** *p* < 0.001.

Figure 5a presents a Principal Coordinates Analysis (PCoA) based on Bray–Curtis dissimilarity at the OTU level, illustrating a clear separation of bacterial communities among the three successional stages—swamp (SP), swamped meadow (SM), and meadow (MW). The first principal coordinate (PC1) accounts for 26.19% of the total variation, while the second coordinate (PC2) explains 23.01%. Figure 5b displays a Non-metric Multidimensional Scaling (NMDS) plot using the same distance matrix, with a stress value of 0.15, indicating a reliable ordination. The NMDS results reveal distinct clustering patterns for each successional stage, consistent with the PCoA findings. To statistically support the observed community differences, a PERMANOVA test based on Bray–Curtis dissimilarity was performed. The results demonstrated a significant difference in microbial community composition among the three stages (R^2^ = 0.5749, *p* = 0.001). Furthermore, pairwise comparisons between all stage pairs remained significant after Bonferroni correction (*p* < 0.05), confirming the robustness and statistical validity of the community separation patterns visualized in both ordination analyses.

### 3.6. Correlation Between Soil Physical and Chemical Properties and the Relative Abundance of Microbial Communities

**Figure 6:**
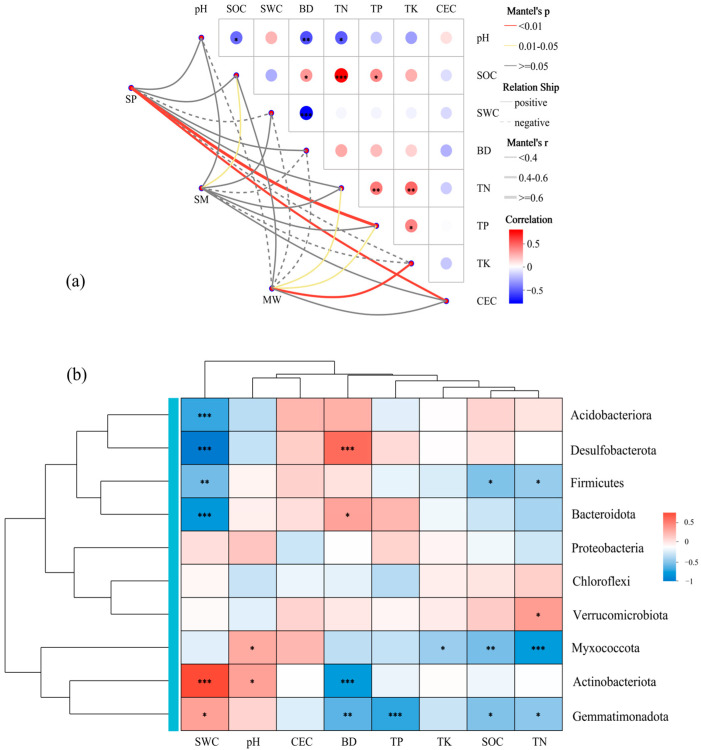
Result from Mantel test (**a**) and Heatmap (**b**) to explore the relationship between the microbial community composition and soil physicochemical properties. Significance levels: * *p* < 0.05, ** *p* < 0.01, *** *p* < 0.001. Abbreviations: SP, swamp; SM, swamped meadow; MW, meadow. pH, soil pH value; BD, soil bulk density; SOC, soil organic carbon; TN, total nitrogen; TP, total phosphorus; TK, total potassium; CEC, cation exchange capacity.

Based on the Mantel test heatmap analysis, the results indicate that environmental factors are significantly correlated with the microbial community composition at different wetland successional stages (Figure 6a). The heatmap reveals that environmental variables such as SOC, soil pH, SWC, and CEC exhibit significant positive correlations with the microbial community structure, with Mantel’s r values mostly ranging between 0.4 and 0.6 (*p* < 0.05). In contrast, the BD shows a significant negative correlation with microbial community composition, with Mantel’s r values ranging from −0.4 to −0.6 (*p* < 0.05). Based on the Spearman correlation heatmap analysis (Figure 6b) along with the Mantel test results, significant correlations were observed between wetland soil environmental factors and the relative abundance of various phyla. Specifically, environmental factors such as soil pH, SOC, SWC, and CEC exhibited significant positive correlations with dominant phyla, including Actinobacteriota, Proteobacteria, and Chloroflexi, while showing significant negative correlations with phyla such as Acidobacteriota, Verrucomicrobiota, and Gemmatimonadota. In addition, the BD was significantly negatively correlated with phyla like Actinobacteriota and Proteobacteria, but significantly positively correlated with Acidobacteriota, Gemmatimonadota, and Verrucomicrobiota. These findings align with the trends observed in the Mantel test heatmap, suggesting that soil physicochemical properties play a crucial role in shaping microbial community structure during different wetland successional stages, with particular emphasis on the close associations of pH, SOC, SWC, CEC, and BD with phylum-level distribution patterns.

### 3.7. Phenotypic Prediction and Functional Analysis

**Figure 7:**
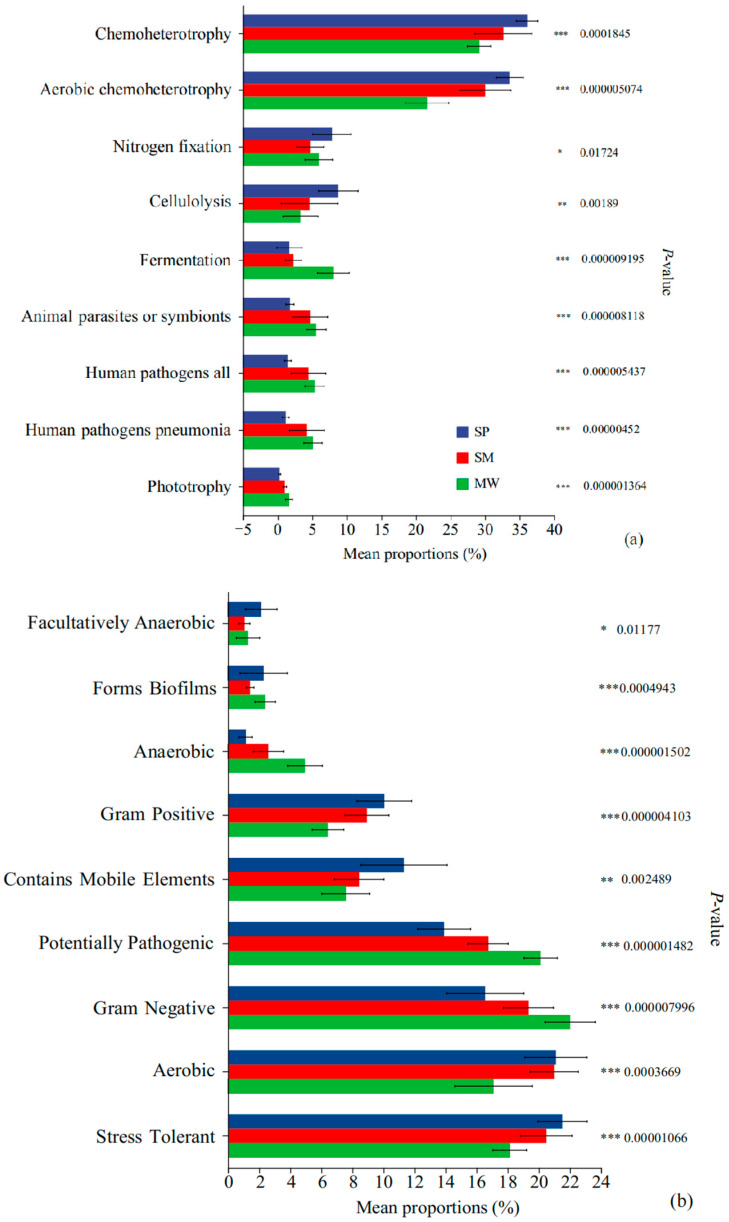
FAPROTAX function prediction (**a**) and BugBase phenotype prediction (**b**) in different succession stages of wetland. Note: significance levels: * *p* < 0.05, ** *p* < 0.01, *** *p* < 0.001.

Based on the FAPROTAX functional prediction analysis (Figure 7a), significant differences in microbial community function were observed among the different wetland successional stages. Specifically, the functional groups of Chemoheterotrophy and Aerobic chemoheterotrophy exhibited the highest relative abundance in the MW stage, which was significantly greater than that in the SP and SM stages (*p* < 0.001). The SP stage showed a significantly higher relative abundance of functions such as Fermentation, Nitrogen fixation, and Cellulolysis compared to the SM and MW stages (*p* < 0.001). Moreover, the SM stage had notably higher abundances of animal parasites or symbionts, human pathogens all, and human pathogens pneumonia relative to the SP and MW stages (*p* < 0.05).

According to the BugBase functional analysis (Figure 7b), significant differences in microbial functional groups were observed among the different wetland successional stages. Facultatively anaerobic microbes showed the highest relative abundance in the MW stage, significantly higher than in the SP and SM (*p* < 0.05). In contrast, microbes associated with biofilm formation were significantly more abundant in the SP stage compared to SM and MW (*p* < 0.001). Similarly, the SP stage also exhibited significantly higher abundances of anaerobic microbes, Gram-positive microbes, and those containing mobile elements (*p* < 0.001). In the MW stage, the relative abundances of potentially pathogenic microbes, Gram-negative microbes, and aerobic microbes were significantly elevated compared to the SP and SM (*p* < 0.001). Additionally, stress-tolerant microbes were most abundant in the SM stage (*p* < 0.01). Overall, these findings reflect marked shifts in microbial functional composition across different stages of wetland succession.

## 4. Discussion

### 4.1. Relationship Between Soil Physicochemical Properties and Diversity

During retrogressive succession in cold-region wetlands, variations in soil physicochemical properties substantially influence microbial diversity, reflecting dynamic and reciprocal feedbacks between soil conditions and microbial communities [36]. Among these, SOC serves as a critical regulator of microbial richness and composition. Elevated SOC provides ample substrates that support diverse microbial metabolisms, resulting in increased values in diversity indices such as Shannon, Chao1, ACE, and Sobs. This enrichment promotes ecological niche differentiation, supporting structurally complex microbial communities [37]. In turn, microbial processes such as organic matter decomposition contribute to SOC turnover and stabilization, thus enhancing soil fertility and ecosystem functionality [38]. Nutrient factors including TN, TP, and TK also significantly contribute to microbial diversity patterns. Increased nutrient availability across succession stages supports microbial growth and functional expansion, reflected in elevated diversity indices [39]. Microbial taxa play a critical role in mediating nutrient transformations such as nitrogen fixation, nitrification, and phosphorus mobilization, thereby shaping nutrient cycling processes and altering plant–microbe interactions [40,41]. The SWC modulates diversity through its influence on soil aeration and redox potential. In earlier succession stages (e.g., SP), high SWC favors anaerobic or facultative anaerobic taxa while potentially suppressing overall community evenness due to limited oxygen-dependent processes [42]. Meanwhile, microbial biofilm formation and extracellular polymer production modify soil structure and water retention, influencing moisture dynamics and microbial habitat quality [43]. The CEC is another key factor that influences microbial diversity. Soils with higher CEC can retain and buffer essential nutrients, mitigating stress and supporting diverse microbial communities [44]. Microbial exudation of organic acids and enhancement of mineral weathering processes further increase CEC, promoting nutrient availability and microbial stability [45]. The BD exhibits a clear negative relationship with microbial diversity metrics such as Shannon and ACE. Increased compaction, especially in MW, restricts soil porosity and oxygen availability, thereby suppressing the proliferation of aerobic microbes [46]. However, microbial activity can mitigate soil compaction by contributing organic matter and altering soil structure, creating more hospitable environments for microbial colonization. Collectively, these findings reveal that interactions between soil physicochemical factors and microbial communities are bidirectional and complex, playing a fundamental role in regulating microbial diversity and ecosystem succession in wetlands [47]. Understanding these interactions is critical for informing conservation strategies, as microbial diversity underpins key ecological processes that support wetland resilience and biogeochemical cycling amid environmental change [48].

### 4.2. Environmental Factors Influencing Soil Microbial Community Structure

In cold-region wetlands undergoing retrogressive succession, microbial community composition responds sensitively to shifts in soil nutrient availability, moisture regimes, and other key physicochemical attributes [49]. These environmental changes reshape microbial assemblages and modulate ecosystem functions, highlighting the interplay between abiotic stressors and microbial adaptation mechanisms [50].

Phylum-level transitions, including the observed shift from Actinobacteria dominance in the SP and SM stages to Proteobacteria predominance in the MW stage, underscore microbial adaptation to changing edaphic conditions. Actinobacteria, typically oligotrophic and tolerant of low-nutrient, high-moisture conditions, are well suited to early successional environments, whereas Proteobacteria thrive in nutrient-rich, oxygenated soils typical of later succession [51,52,53]. Class-level changes, such as reductions in Alphaproteobacteria and Thermoleophilia across succession, suggest functional shifts in nitrogen cycling and organic matter turnover. For instance, Alphaproteobacteria often associated with nitrogen fixation and rhizosphere interactions decline as nutrient availability improves, reflecting a decreased reliance on symbiotic associations [54,55]. Similarly, the decline in Rhizobiales abundance during the transition to MW aligns with reduced demand for nitrogen fixation in increasingly fertile soils [56,57]. At the family level, groups such as Acidobacteriales and Xanthobacteraceae were strongly shaped by pH and SOC conditions. Acidobacteriales, favoring acidic, nutrient-poor environments, decreased in relative abundance with progression in succession, indicating metabolic shifts toward taxa adapted to more favorable chemical conditions [58]. Correlation analyses further support the link between soil factors and microbial distribution patterns. Positive associations of SOC, pH, and CEC with Actinobacteria and Proteobacteria suggest that these taxa benefit from improved nutrient status and buffering capacity [59,60]. In contrast, negative correlations between BD and aerobic microbial groups imply that increased compaction during succession reduces oxygen availability and microbial activity [61,62].

These findings are consistent with recent international and domestic studies, reinforcing the generality of microbial responses to succession in wetland systems [63,64]. However, local environmental variables and biotic interactions may generate site-specific microbial assemblages, demonstrating the flexibility and context dependence of microbial adaptive strategies [65]. Overall, the microbial shifts observed during retrogressive succession reflect both universal and localized responses to changing environmental constraints in cold-region wetlands.

### 4.3. Functional Prediction Analysis of Soil Bacterial Communities in Succession Stages

The predicted functional composition of soil bacterial communities varied significantly across successional stages, reflecting environmental heterogeneity and corresponding microbial adaptations [66]. These shifts imply changes in ecological strategies as microbes respond to differences in nutrient availability, moisture regimes, and redox conditions [67].

According to FAPROTAX predictions, distinct patterns of potential microbial functions were observed. Functions related to chemoheterotrophy and aerobic chemoheterotrophy were predicted to be more prevalent in the MW stage, possibly due to lower water content and higher oxygen availability that favor aerobic metabolism [68]. These functions typically correspond to microbial taxa adapted to nutrient-rich, well-aerated soils found in later succession stages [69]. In contrast, fermentation, nitrogen fixation, and cellulolysis were predicted to be more common in the SP stage, indicating microbial adaptation to saturated and oxygen-limited environments [70]. Additionally, microbial traits associated with animal parasitism, symbiosis, and potential pathogenicity were more frequently predicted in the SM stage, suggesting adaptation to transitional moisture and nutrient conditions [71]. These trends point to functional shifts aligned with environmental changes along the successional gradient [72].

BugBase analysis similarly revealed stage-specific differences in microbial traits. Facultatively anaerobic traits were more frequently predicted in the MW stage, consistent with microbes adapting to variable oxygen and moisture availability [73]. The SP stage showed higher predicted abundances of biofilm-forming, anaerobic, Gram-positive bacteria and taxa containing mobile genetic elements. These features likely aid microbial persistence in saturated soils by enhancing structural stability and resilience to environmental stress [74]. Conversely, MW soils were predicted to support higher levels of aerobic, Gram-negative, and potentially pathogenic microbes, consistent with drier, nutrient-enriched conditions [75]. Stress-tolerant traits were more associated with the SM stage, suggesting microbial community flexibility in fluctuating environments [76].

While these functional predictions provide useful insights into potential microbial responses during retrogressive succession, it is important to recognize that FAPROTAX and BugBase rely on taxonomic inference rather than direct measurement of gene expression or metabolic activity. Therefore, predicted functions should be interpreted as hypotheses, not definitive evidence of microbial roles [77]. Their resolution is limited by the precision of 16S rRNA data and the scope of available functional reference databases [78,79]. Future studies incorporating metagenomics, metatranscriptomics, or quantitative PCR are recommended to validate and refine these predictions.

## 5. Conclusions

This study provides a comprehensive assessment of microbial community structure and functional potential across retrogressive successional stages in cold-region wetlands. Clear differences in taxonomic composition and predicted metabolic functions were observed among swamp (SP), swamped meadow (SM), and meadow (MW) soils, reflecting the strong influence of environmental gradients on microbial ecology. Variations in soil physicochemical properties, particularly SOC, TN, SWC, pH, CEC, and BD, were identified as key drivers shaping microbial communities. Proteobacteria and Actinobacteria were dominant phyla whose relative abundances shifted markedly across succession, indicating niche adaptation to nutrient availability and moisture conditions. Functional predictions revealed that early-stage wetlands supported microbial groups associated with anaerobic metabolism, nitrogen fixation, and cellulose degradation, while later stages were enriched in aerobic chemoheterotrophs. The transitional SM stage exhibited mixed functional traits, including stress-tolerant and facultatively anaerobic microbes, consistent with fluctuating environmental conditions. Notably, mobile genetic elements and biofilm-forming taxa were more abundant in saturated soils, suggesting microbial strategies for adaptation under low-oxygen, high-nutrient conditions. These results highlight the critical ecological roles that microbes play in biogeochemical cycling and wetland resilience during degradation.

To support future restoration and management strategies in cold-region wetlands, the findings underscore the value of microbial indicators for monitoring soil health, hydrological restoration success, and ecosystem functional recovery. For example, the presence of aerobic chemoheterotrophs and stress-tolerant taxa in the meadow and swamped meadow zones may indicate improved aeration and ecological stabilization. Likewise, the detection of mobile genetic elements and facultative anaerobes may serve as early warning signals of persistent water saturation or disturbance stress. While the observed patterns were robust and supported by both taxonomic analyses and multivariate statistics, the relatively limited number of replicates (*n* = 6 per group) may limit the extrapolation of results to broader spatial contexts. Future studies should incorporate larger sample sizes and multiple wetland systems across spatial and temporal gradients to validate these trends and further elucidate microbial ecological strategies under long-term environmental change.

## Figures and Tables

**Table 1:** Physical and chemical properties of soil in different succession stages of wetland.

Variables	SP	SM	MW
pH	6.56 ± 0.30 a	6.57 ± 0.18 a	6.37 ± 0.24 a
SOC (g·kg^−1^)	27.82 ± 1.64 b	25.91 ± 2.69 b	32.35 ± 1.16 a
SWC (%)	91.57 ± 2.43 a	65.22 ± 2.19 b	53.59 ± 5.07 b
BD (g·cm^−3^)	0.90 ± 0.05 b	0.87 ± 0.13 b	1.23 ± 0.07 a
TN (g·kg^−1^)	3.43 ± 0.31 a	2.65 ± 0.10 b	3.71 ± 0.07 a
TP (g·kg^−1^)	0.22 ± 0.13 c	0.25 ± 0.01 b	0.36 ± 0.03 a
TK (g·kg^−1^)	2.53 ± 0.06 b	1.89 ± 0.12 c	3.03 ± 0.11 a
CEC (cmol·kg^−1^)	6.59 ± 0.72 b	10.13 ± 0.86 a	7.71 ± 0.74 b

Note: values are presented as mean ± standard deviation (*n* = 6). Different letters indicate significant differences among successional stages (ANOVA, *p* < 0.05). Abbreviations: pH, soil pH value; SOC, soil organic carbon; SWC, soil water content; BD, soil bulk density; TN, total nitrogen; TP, total phosphorus; TK, total potassium; CEC, cation exchange capacity; SP, swamp; SM, swamped meadow; MW, meadow.

## Data Availability

The datasets generated during and/or analyzed during the current study are available from the corresponding author upon reasonable request.
